# Prevention of Diabetes and Cardiovascular Disease in Obesity

**DOI:** 10.3390/ijms21218178

**Published:** 2020-10-31

**Authors:** Lucia La Sala, Antonio E. Pontiroli

**Affiliations:** 1 Laboratory of Cardiovascular and Dysmetabolic Disease, IRCCS MultiMedica, 20138 Milan, Italy; 2 Dipartimento di Scienze della Salute, Università degli Studi di Milano, 20142 Milan, Italy

**Keywords:** prevention, obesity, diabetes, T2D, CVD, atherosclerosis, inflammation, microRNA, metabolic syndrome, cardiovascular complications

## Abstract

Obesity is one of the major risk factors for the development of both impaired glucose tolerance (IGT, or prediabetes) and type 2 diabetes (T2D), and its prevalence worldwide drives toward an increased rate of cardiovascular morbidity and mortality. Given the estimations of the World Health Organization (WHO) and the recommendation of the Diabetes Prevention Program (DPP), where IGT and diabetes are considered as risk factors for the development of cardiovascular complications and obesity, the development of diabetes should be treated because of its potential reversibility. In this view, several interventions such as diet, lifestyle changes, and pharmacological treatment are effective, including bariatric metabolic surgery (BMS), which is the most incisive way to efficiently lower body weight. In this review, we sought to summarize some of the major aspects linked to diabetes prevention in overweight/obesity, focusing on the use of surgery; we also attempted to elucidate molecular pathways involved in a variety of obesity-induced processes able to favor the progression of chronic diseases, such as diabetes and its complications.

## 1. Introduction

The prevalence of obesity has risen dramatically worldwide over the last decades, reaching epidemic proportions. It has been estimated by the World Health Organization (WHO) that the number of obese adults has increased more than 7-fold in the last 40 years [1]. Obesity is associated with a high prevalence of impaired glucose tolerance (IGT, or prediabetes), and it is an independent risk factor for type 2 diabetes (T2D) [2], as shown by data in the general population and in patients who are candidates for bariatric surgery [3,4]; in turn, prediabetes is associated with the risk of progression to diabetes, and it has been recognized for a long time that prediabetes unfavorably affects the cardiovascular system [5]. Growing evidence has shown a dramatic increase in overweight/obesity prevalence also among adolescents. Recently, a cohort study on an adolescent population has evidenced that increased body mass index (BMI) is associated with a higher risk to develop T2D in the adult life [6]. Other data regarding the reversibility of obesity in adolescents or young adults that in childhood were obese have focused on the reduction of the risk of developing T2D in adulthood [7]. Given these premises, it appears of utmost importance in obesity to prevent progression to diabetes, and in the meanwhile to treat co-morbidities of obesity, which are mainly represented by T2D, metabolic syndrome (MetS, a group of disorders strictly linked with each other and associated with insulin resistance (IR), elevated levels of triglycerides, and finally hyperglycemia), and cardiovascular disease (CVD). The Diabetes Prevention Program (DPP) recommended prevention programs as hints to reduce the risk to develop T2D, including adopting healthy habits (diet and physical activity) and avoiding smoking, alcohol, and stress. In fact, intensive lifestyle intervention was able to reduce the incidence of T2D by 58% over 3 years [8,9]. Therefore, preventing T2D in obesity seems to be critical for preventing metabolic diseases and cardiovascular complications.

## 2. Pathogenesis of Diabetes in Obesity

**Figure A1:**
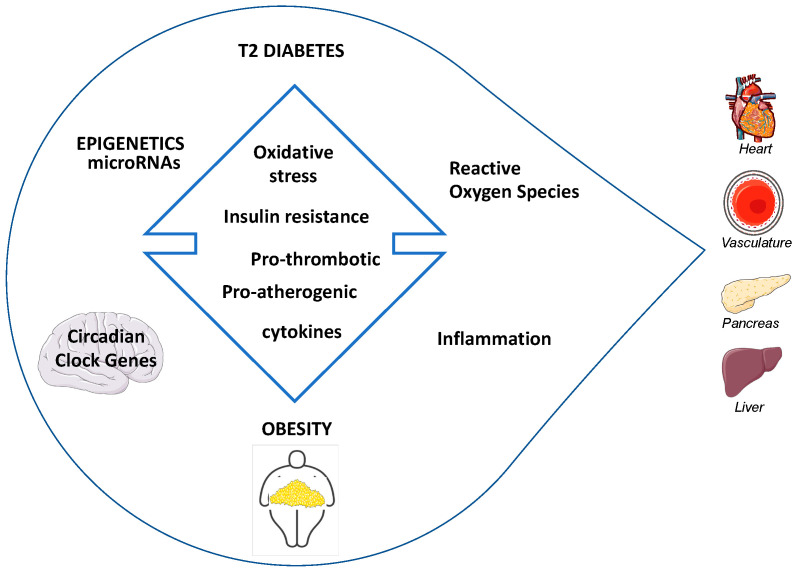
Major contributors of T2D/Obesity driving toward to dysregulation of target tissues.

The pathogenesis of diabetes and obesity is similar, sharing common pathways of IR, oxidative stress (Ox-S), and pro-thrombotic and pro-inflammatory patterns [10,11]. The obesogenic environment, stimulating overnutrition, leads to dysregulation of the metabolic balance and subsequent fat accumulation in organs that are not specialized in lipid storage (ectopic fat), such as the endothelium, the liver, and the skeletal muscle, inducing metabolic disturbances and disorders, such as IR, IGT, and T2D, cardiovascular diseases, cerebrovascular diseases, and liver diseases (Figure A1). Several mechanisms are strictly linked with the onset of CVD and atherosclerosis, in which endothelial dysfunction plays a major role [12].

### 2.1. Non-Esterified Fatty Acids (NEFAs) and Nitric Oxide (NO)

From an endothelial perspective, in obese people, the low levels of nitric oxide (NO) bioavailability lead to an impairment of endothelium-dependent vasodilation [13,14], with the consequent alteration of endothelial nitric oxide synthase (eNOS) and enhancement of Ox-S, inflammation, and atherosclerotic burden [11]. The mechanisms through which obesity leads to IR and subsequently to T2D are not fully understood. Several studies have shown in obesity that adipose tissue releases high amounts of circulating non-esterified fatty acids (NEFAs), hormones, and pro-inflammatory cytokines, which drive to IR by the inhibition of glucose transport and phosphorylation [15]. The increase in NEFAs provides the basis for the activation of the serine/threonine kinase cascade (induced by fatty acid metabolites such as ceramides, diacylglycerol, or fatty-acyl coenzyme A), leading to the phosphorylation of insulin receptor substrates 1 and 2 (IRS-1 and IRS-2). The ability of these receptor substrates can activate the phosphoinositide 3-kinase (PI3K)/protein kinase B (AKT)/eNOS pathway, reducing insulin receptor signaling and hence glucose transport. A hyperglycemic milieu could be worse from an endothelial perspective, especially in compromising the binding with the insulin receptor, activating a transduction way in favor of pro-atherogenic effects instead of anti-atherogenic responses. De Nigris et al. showed that under high levels of glucose and insulin-stimulated endothelial cells, the pro-atherogenic pathway seems to be more prone to activation [16]. In fact, the phosphorylation of eNOS at Ser1177, activated by AKT kinase, leads to the production of NO; but it also activates the phosphorylation of Src-homology 2 domain-containing (SHC) transforming protein, which affects the activation of the mitogen-activated protein kinase (MAPK) pathway, resulting in increased endothelin-1 (ET-1) expression and mitogenic effects [17].

### 2.2. Clock Genes

A piece of further evidence underlying the onset of T2D in obesity is represented by the alteration of the so-called “clock genes”, which are considered as the precursors in the development of other metabolic complications. Circadian clocks are a group of genes responsible for coding the proteins needed for the generation and regulation of circadian rhythms, as demonstrated in both rodents [18] and humans [19]. The shift in circadian clock genes increases the risk of metabolic disorders, particularly obesity and IR. Several studies investigated the influence of the disturbance of clock gene expression on the development of obesity and diabetes. Various parameters related to glucose metabolism, such as glucose tolerance, insulin sensitivity, as well as glucose, glucagon, and insulin plasma levels are known to exhibit circadian variations throughout the day [20]. A dysregulation in the *Circadian Locomotor Output Cycles Kaput (CLOCK)* gene is able to disrupt the rhythm in feeding, promoting inactivity, enhancing hyperphagia, hyperlipidemia, hyperglycemia, and hypoinsulinemia [21]. Further studies focused on the *Brain and Muscle Arnt-Like protein-1 (BMAL1)* gene, which is a critical circadian transcription factor that encodes for protein Arnt-Like (ARNTL) forming a heterodimer with CLOCK. Mice lacking BMAL1 showed an alteration of adipogenesis and hepatic carbohydrate metabolism [22]. Otherwise, mice with inactivated BMAL1 showed a suppressed diurnal variation in glucose and triglycerides, which led to impaired gluconeogenesis and recovery from insulin-induced hypoglycemia [23]. In insulin-resistant mice, the perturbation of CLOCK, BMAL1, reduced c-erb-αoncogene (REV-ERBα) and Cryptochrome Circadian Clock 1 (CRY1) mRNA expression is evident during obesity [24]. Indeed, it seems that an activation of BMAL1, PPARα-induced, might have a direct link with high concentrations of retinoic acid (increased in metabolic syndrome [25]) and reduced REV-ERBα levels in obesity and IR [24]. The role of the transcription factor *REV-ERBα* gene, associated with MetS, was demonstrated by Vieira et al. in a study investigating clock gene expression in visceral adipose tissues (VATs) in lean and obese female subjects [26]. Furthermore, elucidating the role of clock genes might be helpful in the design of therapeutic interventions for obesity and T2D. For instance, the use of REV-ERB agonists in diet-induced obese mice increased energy expenditure, reduced fat mass, and improved dyslipidemia and hyperglycemia [27]. Furthermore, it has been proved that REV-ERB agonists would be beneficial in prevention or treatment models for atherosclerosis of LDL receptor null mice [28]. Due to its chemistry, REV-ERB ligands would be a target for the treatment of metabolic disease; thus, since high levels of REV-ERB have been correlated with increased insulin secretion and reduced glucose concentration, the use of REV-ERB agonists would be a potential treatment for T2D.

### 2.3. Insulin Resistance (IR) and Reactive Oxygen Species (ROS)

Obesity and T2D are closely associated with insulin resistance (IR) and the development of cardiovascular disorders (micro- and macrovascular), whose central factors are conveyed into processes of Ox-S [11]. Some studies have shown the role of Ox-S in the hyperglycemic phenotype and in a model of glucose variability using endothelial cells [29,30,31,32]. The glucose-derived cellular damage has been related to a hyperglycemia-induced overproduction of mitochondrial reactive oxygen species (ROS), resulting in defective ROS homeostasis and in the inactivation of antioxidant responses, in particular superoxide dismutase-2 (SOD-2) and glutathione peroxidase-1 (GPx-1), which are responsible for controlling the rate of radicals produced under stress conditions.

Obesity is well known to be affected by Ox-S having a higher susceptibility to activate oxidative pathways dysregulated by concurrent defective ROS scavenging and decreased mitochondrial function. The redox-sensitive transcription factor Nuclear Erythroid 2 related factor-2 (NFE2L2 or NRF2) regulates antioxidant response elements (AREs). In a diabetic milieu, the activity of NRF2 is reduced, contributing to increased Ox-S, mitochondrial dysfunction in vessels, and thus enhancing endothelial dysfunction, as observed in diabetes [33]. NRF2 activation is associated with the prevention of many types of human diseases, including diabetes and obesity [34]. In diabetic complications, elevated levels of circulating markers of lipid peroxidation were observed, reflecting the oxidative damage in several tissues [35].

### 2.4. Insulin Resistance (IR) and Inflammation

Both obesity and diabetes, in their pathogenesis and in the development of complications, share a common phenotype characterized by IR and the activation of inflammatory processes [36,37]. Many key inflammatory markers have been associated with both obesity and the risk of adverse outcomes in obesity-associated diseases, and this suggests that a persistent, low-grade inflammatory response is a potentially modifiable risk factor. The relationship between adipose tissue inflammation and glycemic control is complex. In fact, dysfunctional adiposity is characterized by an altered gene expression profile in the context of obesity and type 2 diabetes, which is not easy to identify [38].

An inflammatory role has been hypothesized in the pathways that drive toward the progression from obesity to diabetes. As demonstrated by an ARIC (Atherosclerosis Risk in Communities) study, a case-cohort study performed on diabetic and non-diabetic middle-aged subjects, inflammatory factors (a panel of six markers) have a major role in the pathways that lead to the progression from obesity to diabetes; in this population, obese individuals had a more than 6-fold higher risk of developing diabetes [39]. In ARIC, NEFAs was an independent predictor of diabetes and thus might play a mediating role; the hypothesized mechanisms focused on the involvement of an impaired insulin signaling pathway. In obese rats, the peripheral uptake of glucose in response to insulin was induced by the reduction of a pro-inflammatory cytokine, tumor necrosis factor-alpha (TNF-α) activity [40]. The underlying mechanism of the induction of IR by TNF-α has two different pathways:

1. the activation of transcription factor NF-κB, which is involved in insulin-sensitivity, and Inhibitor of Nuclear Factor Kappa B Kinase Subunit β (IKKβ), and
2. by directly inhibiting IRS-1 with phosphorylation on its serine residues [41].

Thus, the activated NF-κB promotes the transcription of multiple inflammatory mediators, such as TNF-α and interleukin-6 (IL-6) [42], promoting inflammation-induced IR pathway.

Furthermore, in non-obese diabetic (NOD) mice, autoimmunity has a prominent role, since inflammation can trigger the recruitment and activation of dendritic cells/macrophages, leading to the disruption of β-cells [43]. Finally, a possible role of resistin might be considered as a new link between obesity and diabetes, since resistin levels are increased in diet-induced obesity, as well as in genetic models of obesity and IR, and its inhibition enhanced insulin-stimulated glucose uptake [44]. Researchers also demonstrated the ability of the NOD-like receptor pyrin domain-containing protein 3 (NLRP3) inflammasome, in which mRNA expression in VAT was correlated with body weight, to alter insulin sensitivity and IR through the processing of IL-1β and IL-18 in response to obesogenic stimuli as LDL, hypoxia, and ROS, promoting a chronic pro-inflammatory state [45].

## 3. Progression of IGT to T2D in Obesity: From Epigenetics to the Role of miRNAs

The prevalence of diagnosed type 2 diabetes mellitus continued to increase concurrently with increases in obesity. Several experimental studies have highlighted the role of epigenetics in the control of progression toward pre-/diabetes. Mechanisms such as the methylation of genes and expression of non-coding small RNA molecules (microRNAs) play a crucial role in these processes. MicroRNAs (miRs), a single-stranded small molecule of non-coding RNA highly conserved among species and regulating gene expression (as summarized in [46]), might be related to the progression of prediabetes toward diabetes: in the DIAPASON (Diabetes Prediction and Screening Observational) study (detailed in [47]), it was possible to identify IGT progressors vs. non-progressors, predicting diabetic progression in the prediabetic population [48]. Parrizas et al. proposed both miR-192 (which modulates adipocyte differentiation and lipid homeostasis in obese subjects [49]) and miR-193b (which modulates adiponectin production in white adipose tissue (WAT) and therapeutic target for IR [50]) as markers of prediabetes. Indeed, the downregulation of miR-192 only in diabetics suggested a possible therapeutic role to prevent diabetes [51]. As aforementioned, miRs are non-coding RNA molecules of 25 nucleotides in length that are able to modulate gene expression, and they are emerging as new biomarkers for predicting diseases. A set of 24 circulating miRs selected by nutritional therapy based on a low-fat–high complex carbohydrate diet (LFHCC) recommended by the American Heart Association (AHA) have emerged as preventive for T2D development in subjects with CVD [52]. In addition, miR-21 in T2D subjects might be used as a novel biomarker induced by elevated ROS that exert their dangerous activity on lipid peroxidation [31].

### 3.1. Novel miRNA-Based Approaches for the Prevention of Diabetes Using Bariatric Metabolic Surgery (BMS)

Bariatric metabolic surgery (BMS) has emerged as potentially useful for morbid obesity. BMS leads to a remission/resolution of T2D, improving glycemic control [53], insulin sensitivity, hyperlipidemia, and other obesity-associated disorders, but its precise mechanisms are not yet fully understood. Recently, it has been demonstrated that the beneficial effects of BMS might be regulated by microRNAs that, due to their ability to bind the 3’-UTR of the mRNA target, modulate gene expression [54]. miRNAs have been related with the molecular changes typical of excessive adiposity, which could be resolved by weight loss after BMS. Analyzing the miRNome from obese and normal-weight individuals, some obesity-related changes in adipose tissue in the miRNome were observed: five miRs (hsa-miR-146b-3p, hsa-miR-146b-5p, hsa-miR-223-3p, hsa-miR-223-5p, and hsa-miR-941), which were increased in obese subcutaneous adipose tissues (SAT-O), were significantly reduced after BMS [55].

Recently, in plasma and liver samples of both bariatric patients and a rodent model of BMS, it has been shown that a 90% downregulation of miR-122 and a reduction of miR-342-3p, miR-320, miR-139-5p and miR-146a might regulate metabolic processes such as the citric acid cycle (TCA) cycle, glucose transport, pentose phosphate pathway, fatty-acid synthesis, mitochondrial oxidation, gluconeogenesis, and glycolysis [56,57]. In addition, it has been demonstrated that BMS also altered the content of circulating exosomal microRNAs in obese subjects [57,58]. Furthermore, Alkandari et al. hypothesized that BMS modulates the levels of microRNAs (miR-192 and miR-200), which were found highly expressed in urine before and after surgery in comparison to non-surgical control subjects [59]. Moreover, miR-448 and its target gene SIRT1, the latter associated with cellular metabolism, can serve as prognostic indicators for obese and T2DM patients after BMS [60]. These studies evidenced the role of microRNAs in understanding the landscape of the crosstalk between miRNAs and metabolic pathways that point to a systemic regulation of processes including multiple axes of energy metabolism. In the future, microRNAs might be useful for facilitating decisions about surgery and/or predicting weight loss after bariatric surgery, providing targets for future treatments.

### 3.2. Epigenetics as a Driving Force Controlling Obesity-Related Genes Toward Diabetes

Obesity and physical inactivity, probably in the background of genetic predisposition [2,61], are recognized as major risk factors for diabetes and for the progression of prediabetes to T2D. Longitudinal studies have shown in subjects with long-standing obesity the parallel progression from normal glucose tolerance to prediabetes and from prediabetes to diabetes. In a study on the developmental trajectories of BMI from childhood into late adolescence and subsequent late adolescence–young adulthood cardiometabolic risk markers, BMI trajectory was strongly associated with HDL cholesterol and IL-18 in males and diastolic blood pressure and IL-6 in females. Thus, BMI trajectory was associated with cytokine levels in both sexes. This shows that BMI trajectory confers additional cardiometabolic risk beyond age-specific BMI and it suggests a strong impact of BMI trajectory on pro-inflammatory markers; the sex-specific trajectory–cardiometabolic risk marker association appears noteworthy [62].

FTO (fat mass and obesity-associated), a gene associated with the common forms of obesity [63] and with IR in T2D [64], modulates insulin activity and BMI [65]. FTO regulates the amount of fat deposition and affects T2D risk through its effect on BMI; indeed, people homozygous for a particular FTO allele weighed about 3 kg more and had a 1.6-fold greater rate of obesity than those who had not inherited this trait. At the molecular level, the driving force for the activation of pro-inflammatory genes is traceable to the epigenetic processes as the chromatin arrangements that are deputed to control gene expression in the metabolic processes and in nutritional requirements; this view would be important for stimulating structural adaptations driving to deleterious consequences on the co-morbidities of obesity, including diabetes and CVD. As other pathologies, an obesogenic environment prone toward diabetes favors the addition of a methyl group to DNA, which is mediated by methyltransferases (DNMTs), representing the critical regulators for the stimulation of pro-inflammatory cytokines, obesity-induced, to selectively methylate and stimulate the compacting chromatin structure in the gene promoter, and thus exacerbating IR [66]. However, the methylation levels in the genes involved in lipid metabolism, such as lipoprotein lipase (LPL) essential in storing or consuming triglycerides, are altered in obese patients with the metabolic disease, indicating a difference from healthy people [67]. In a recent work, a significant influence of fats on site-specific DNA-methylation (DNA-me) relevant to T2D was found [68]; for instance, the associations between high levels of DNA-me in CpG (or CG island) site of a gene essential in cholesterol transport (*ABCG1*) correlated with fasting insulin and HOMA-IR in CD4^+^ T cells from non-diabetic individuals [69], suggesting that the derangements of the metabolic state that drive to T2D onset could be influenced by DNA-me on the obesogenic genes. Since genetic variations have occurred for the development of obesity and diabetes, genome-wide associations studies (GWA) have highlighted various genes, e.g., the *melanocortin-4 receptor (MC4R)* gene for obesity [70] and peroxisome proliferator-activated receptor gamma *(PPARG)* gene [71] and *islet potassium voltage-gated channel subfamily J member 11 (KCNJ11)* gene [72] for T2D. More than 150 genetic loci are involved in the development of obesity and diabetes [73]. In addition, associated with adipocyte function, the gene encoding KLF14 (Krupper-Like Factor 14) appears as a key player in human metabolism being associated with IR [74,75] and the development of prediabetes. It appears more interesting that in humans, hypocaloric dietary intervention changed the methylation levels of obesity-related genes in obese people [76], ameliorating the phenotype; also, in mice [77], an earlier hypocaloric dietary treatment dramatically increased the DNA methylation status.

## 4. Obesity as a Major Risk Factor for Cardiovascular Disease

The interplay of obesity, severe obesity, insulin resistance, and chronic inflammation and the development of cardiac diseases has been highlighted [78]. Obesity has been recognized as a risk factor for heart failure (HF) [79], coronary heart disease (CHD), and premature death [80,81]. The CVD risk factors related to obesity (e.g., elevated cholesterol and high blood pressure) have a high prevalence in overweight and obese people, even though the increased use of medications has reduced risk factors for CVD and improved disease management. Many studies have focused on the influence of different hormones and circulating factors such as adipokines, chemokines, and growth factors on the inflammatory component and prothrombotic effects, considering them the basis of the connection between obesity and cardiovascular diseases [82].

The importance of the tight control of blood glucose in either preventing or delaying the progression of diabetes complications is now recognized [83,84]. Recent longitudinal studies indicate an increased risk for cardiomyopathy, HF, cardiovascular mortality, and all-cause mortality in adulthood for adolescents with severe obesity compared to those with mild obesity [85]. Given the alarming increase in the prevalence of severe obesity, the persistence of adiposity from childhood to adulthood and the precarious course of young adults with chronic comorbidities deserves attention. Normal-weight central obesity in women was associated with an excess risk of mortality, similar to that of women with BMI-defined obesity with central obesity [86]. These findings underscore the need for future public health guidelines to include the prevention and control of central obesity, even in individuals with normal BMI. None of the approaches identified any obese subgroup free of increased risk of cardiovascular events compared with normal-weight healthy participants. A benign obese phenotype might be defined by strict definitions, but insufficient studies exist to support this. More research is needed to better define metabolically healthy obese (MHO) [87].

Dynamic changes in metabolic health and obesity status were observed during a relatively short interval of 3–5 years. A loss of metabolic health was significantly associated with an increased hazard of HF. Even if metabolic health was maintained, persistent obesity remained a risk factor for HF, while leaning (transition from MHO to MHNO (metabolically healthy non-obese)) had a protective effect against HF. Therefore, the prevention and control of obesity while maintaining metabolic health would be crucial in preventing HF [88]. Myocardial microvascular function gradually declined with increasing BMI in both diabetes and non-diabetes status. T2D was associated with an increased risk of microvascular dysfunction, and obesity exacerbated the adverse effect of T2D [89].

Obesity is highly prevalent among patients with hypertrophic cardiomyopathy and is associated with an increased likelihood of obstructive physiology and adverse outcomes. Strategies aimed at preventing obesity and weight increase may play an important role in the management and prevention of disease-related complications [90]. Prediabetes is associated with structural right atrial and right ventricle changes as well as impaired right ventricle systolic and diastolic function, independent of cardiovascular risk factors. These associations were largely not mediated by indices of left ventricle structure, left ventricle function, or pulmonary pressure. This suggests that (pre)diabetes affects the right atrial and right ventricle structure and function due to direct myocardial involvement [91]. Weight changes of more than 10% after diabetes diagnosis were associated with higher mortality, and over 10% weight gain was associated with an increased risk of stroke [92].

Three aspects of obesity deserve discussion. One is the concept of the obesity paradox, emerging from cross-sectional studies, in which overweight/obesity is associated with a favorable outcome in various cardiovascular diseases such as IMA and HF, although the paradox may reflect an epidemiological artifact rather than a true negative association between normal weight and clinical outcomes [93]. However, intentional weight loss carries a lower risk of death [94]. The other concept is metabolically healthy obesity; the issue is ill-defined, and discussion is ongoing as to the fact that metabolically healthy obesity is a true entity or a momentary phase in the development of full-blown obesity [95]. Finally, the exact role of central obesity and visceral obesity in the development of cardiovascular disease is far from being ascertained [96]. Other explanations are possible; in a cohort study of 1 million men on cardiorespiratory fitness and obesity in adolescence, and later chronic disability due to cardiovascular disease, it was found that overweight/obesity was associated with CVD disability for all investigated causes, suggesting associations between low levels of cardiorespiratory fitness and obesity with later risk of chronic disability due to CVD [97].

## 5. Available Means to Prevent the Progression of Prediabetes to Diabetes: Lifestyle, Pharmaceutical, and Other Approaches

Many approaches have been proposed to fight the obesity epidemics, usually recommending a combination of diet and/or physical activity with behavioral support, including BMS and medications. Various strategies are available to prevent the progression of prediabetes to diabetes in obesity at least in the short term, being based on exercise and diet, pharmacologic, and miscellaneous interventions. As shown in at least two meta-analyses, the most effective approach to prevent the progression of prediabetes to diabetes during obesity is represented by a significant loss of weight, which is usually associated with BMS [98,99]. In virtue of the increasing prevalence of obesity and associated co-morbidities, including IGT and T2D mellitus, novel treatment strategies are needed. The multifactorial aspect of obesity (genetic background, metabolic, behavioral, or environmental) for the development of anti-obesity treatments is confusing. Recently, novel classes of antidiabetic therapy, such as sodium-glucose cotransporter (SGLT)-2 inhibitors and glucagon-like peptide-1 receptor agonist (GLP1-RA), have shown anti-obesogenic effects, alone or in combination, and they also are able to exert beneficial effects on atherosclerosis and CVD risk [100]. However, anti-diabetic drugs have many pleiotropic effects, which are responsible for their impact on cardiovascular disease.

SGLT-2 inhibitors (dapa-, cana- and empa-glifozin) reduce plasma glucose levels by inhibiting glucose and sodium reabsorption in the kidneys, thus resulting in glucosuria; GLP1-RAs (e.g., exenatide, liraglutide, and semaglutide) reduce body weight by a secondary effect on satiety, involving many organs (brain, cardiovascular system, duodenum, and kidney, to mention only a few). The effects of both drugs in reducing weight and fat deposition, specifically about ≈2.8 kg [101] and ≈4.5 kg [102,103] in obese individuals without diabetes, are mainly attributable to a loss of subcutaneous (SAT) and visceral adipose tissue (VAT).

More recent studies have shown the possible benefits of liraglutide, a GLP1-RA, compared to placebo [104], and of a combination of linagliptin (belonging to the inhibitors of dipeptidyl peptidase-4 (DDP-4) pharmaceutical class), metformin, and lifestyle on reduced T2D incidence in subjects with prediabetes as compared to metformin and lifestyle [105].

The attempts to prevent diabetes by contrasting inflammation are of interest. Despite favorable anticipations [106], the prevention/treatment of diabetes through pure anti-inflammatory agents has not been successful [107]. However, metformin itself, among its pleiotropic effects, is characterized by anti-inflammatory activity [108], and the role of metformin in prevention–treatment of diabetes/metabolic syndrome is acknowledged; also, sitagliptin with/without vitamin D3 exerts anti-inflammatory activities [109].

In comparison with best medical treatment, BMS is able to induce a remission of clinically overt diabetes [110], together with the improvement of cardiovascular performance (obstructive sleep apnea, left ventricular hypertrophy, hypertension, markers of early atherosclerosis), kidney function, liver function, endothelial dysfunction, and inflammation [111,112].

At least for the prevention of progression to diabetes, several mechanisms are involved; in a tentative list, we should include a reduction of insulin resistance for all BMS, together with a decrease of body fat, and the overall percentage of visceral fat [113]; for Roux-en-Y (RYGB) and for biliopancreatic diversion (BPD), additional factors might be involved, such as an increased secretion of gastrointestinal hormones, not directly related to weight loss, with the possible improvement of insulin release [114,115].

## 6. Long-Term Prevention of Diabetes and Cardiovascular Disease in Obesity

Long-term interventions with 10–20 years follow-up have been able to consistently prevent diabetes, but they have not prevented CV events or the development of micro-angiopathic complications; only the Da Qing study, with a follow-up of 30 years, showed benefit in the prevention of CV diseases [116,117,118,119]. At any rate, the prevention of CV events was observed only in subjects with a significant reduction of body weight. The treatment of obese patients without overt type 2 diabetes with a high dose of liraglutide (Table 1) for a short time induced changes in lipid–lipoprotein and hormonal profile that are suggestive of a lower risk of atherosclerosis and CVD [120]. In contrast, BMS has shown able to prevent diabetes, to prevent micro- and macro- angiopathic diabetic complications, and to prevent CV events [121,122,123,124,125], whatever the age of patients, be it below or above the median age of 43 years [125]. This applied to the reduction of new incident cases of diabetes, CVD, cancer, and impaired kidney function as well as a reduction of hospital admissions for most of the above. The prevention of micro-angiopathic diabetic complications was present in all subsets of subjects with different degrees of glucose tolerance, but it was at its maximum in subjects with prediabetes [122], suggesting that prediabetes should be treated aggressively to prevent future microvascular events.

## 7. Long-Term Prevention of Mortality in Obesity

Only one study showed a prevention of mortality in T2DM through intentional weight loss, as compared to unintentional weight loss or weight gain [126]. Compared to medical treatment, bariatric surgery has consistently been associated, in the long run, in studies up to 23 years, with fewer deaths [127,128]. This has been shown for cardiovascular mortality, mortality due to diabetes, and mortality due to cancer, while mortality due to external causes (i.e., accidents and suicide) was greater in surgery patients than in controls. This reduction of mortality was observed with various kinds of bariatric surgery, with no preference for a given surgical technique, suggesting that weight loss and the prevention of diabetes and CVD are the driving forces in a reduction of mortality [128,129]. However, different from the incidence of new cases of diabetes, which was similar in patients below or above 43 years of age, a reduction of mortality was significant for patients aged more than 43 years but not for younger patients; this was observed first in three studies [124,128,129,130] and confirmed in a meta-analysis [131], suggesting that the difference of efficacy of BMS vs. diet is significant when health conditions are more severe.

## 8. Conclusions

Obesity is a major risk factor for the progression of prediabetes to diabetes, and it confers a risk of cardiovascular disease in both prediabetes and in diabetes. In the last few years, great knowledge has accumulated on the links of obesity with prediabetes/diabetes, especially at the molecular level. In clinical practice, a need to better phenotype the obesities is needed in order to focus the treatment for which effective weight loss and subsequent weight maintenance are of particular importance, as reported in the European Association for the Study of Obesity (EASO) statement [132,133].

The prevention of progression of prediabetes to diabetes is now possible through different approaches, and the main goal remains weight loss. Lifestyle modifications, some drugs, and bariatric/metabolic surgery have a significant effect on weight loss and therefore on prevention of the progression of prediabetes to diabetes; the long-term efficacy of these approaches is different, as bariatric/metabolic surgery is also able to prevent cardiovascular damages in the long run, and to prevent overall mortality. This possibility has not yet been shown for other approaches.

## Figures and Tables

**Table 1:** Established effects of different approaches in prevention of diabetes.

	Prevention of T2D	Prevention of CVD	Prevention of Mortality	Prevention of Complications of Diabetes	Other Preventions (Cancer, Kidney Disease)
Diet	+	not shown	not yet shown	not yet shown	not yet shown
Drugs	++	liraglutide	not yet shown	not yet shown	not yet shown
Surgery	+++	++	++	++	++

+ some effect; ++ good effect; +++ strong effect.

## Abbreviations

ARIC	Atherosclerosis Risk in Communities
ARNTL	Aryl Hydrocarbon Receptor Nuclear Translocator Like
BMI	Body Mass Index
BMS	Bariatric Metabolic Surgery
CLOCK	Circadian Locomotor Output Cycles Kaput
CpG	Cytosine Guanine island
CVD	Cardiovascular Disease
DPP	Diabetes Prevention Program
FTO	Fat Mass and Obesity-Associated Protein
GWA	Genome Wide Association
HF	Heart Failure
IGT	Impaired Glucose Tolerance
IKKβ	Inhibitor of Nuclear Factor Kappa B Kinase Subunit β
IL-6	Interleukin 6
IR	Insulin Resistance
IRS-1	Insulin Receptor Substrate 1
IRS-2	Insulin Receptor Substrate 2
KCNJ11	KATP Channel
KLF14	Krupper-Like Factor 14
MC4R	Melanocortin 4 Receptor
MetS	Metabolic Syndrome
miR	MicroRNA
NF-κB	Nuclear Factor kappa B
NLRP3	Nod-Like Receptor Family Pyrin Domain Containing 3
NOD	Non-Obese Diabetic
T2D	Type 2 Diabetes
TNF-α	Tumor Necrosis Factor
VAT	Visceral Adipose Tissue
WHO	World Health Organization
